# A comprehensive genetic map of sugarcane that provides enhanced map coverage and integrates high-throughput Diversity Array Technology (DArT) markers

**DOI:** 10.1186/1471-2164-15-152

**Published:** 2014-02-24

**Authors:** Karen S Aitken, Meredith D McNeil, Scott Hermann, Peter C Bundock, Andrzej Kilian, Katarzyna Heller-Uszynska, Robert J Henry, Jingchuan Li

**Affiliations:** CSIRO Plant Industry, Queensland Bioscience Precinct, 306 Carmody Rd, St Lucia, QLD 4067 Australia; BSES Limited, Meiers Road, Indooroopilly, QLD 4068 Australia; Southern Cross Plant Science, Southern Cross University, Military Rd, Lismore, NSW 2480 Australia; Diversity Arrays, Technology Pty Ltd, PO Box 7141, Yarralumla, ACT 2600 Australia; Queensland Alliance for Agriculture and Food Innovation, University of Queensland, Brisbane, QLD 4072 Australia

**Keywords:** *Saccharum*, Polyploid, Genetic mapping, SNP markers, DArT sugarcane array

## Abstract

**Background:**

Sugarcane genetic mapping has lagged behind other crops due to its complex autopolyploid genome structure. Modern sugarcane cultivars have from 110-120 chromosomes and are in general interspecific hybrids between two species with different basic chromosome numbers: *Saccharum officinarum* (2n = 80) with a basic chromosome number of 10 and *S. spontaneum* (2n = 40-128) with a basic chromosome number of 8. The first maps that were constructed utilised the single dose (SD) markers generated using RFLP, more recent maps generated using AFLP and SSRs provided at most 60% genome coverage. Diversity Array Technology (DArT) markers are high throughput allowing greater numbers of markers to be generated.

**Results:**

Progeny from a cross between a sugarcane variety Q165 and a *S. officinarum* accession IJ76-514 were used to generate 2467 SD markers. A genetic map of Q165 was generated containing 2267 markers, These markers formed 160 linkage groups (LGs) of which 147 could be placed using allelic information into the eight basic homology groups (HGs) of sugarcane. The HGs contained from 13 to 23 LGs and from 204 to 475 markers with a total map length of 9774.4 cM and an average density of one marker every 4.3 cM. Each homology group contained on average 280 markers of which 43% were DArT markers 31% AFLP, 16% SSRs and 6% SNP markers. The multi-allelic SSR and SNP markers were used to place the LGs into HGs.

**Conclusions:**

The DArT array has allowed us to generate and map a larger number of markers than ever before and consequently to map a larger portion of the sugarcane genome. This larger number of markers has enabled 92% of the LGs to be placed into the 8 HGs that represent the basic chromosome number of the ancestral species, S*. spontaneum*. There were two HGs (HG2 and 8) that contained larger numbers of LGs verifying the alignment of two sets of *S. officinarum* chromosomes with one set of *S. spontaneum* chromosomes and explaining the difference in basic chromosome number between the two ancestral species. There was also evidence of more complex structural differences between the two ancestral species.

**Electronic supplementary material:**

The online version of this article (doi:10.1186/1471-2164-15-152) contains supplementary material, which is available to authorized users.

## Background

Sugarcane is widely cultivated in tropical and subtropical regions and is primarily grown for sugar production accounting for about 75% of the world’s sucrose supply. It is clonally propagated and has a very high photosynthetic efficiency which makes it very attractive as a source of biomass [1]. Recently it has become more important as a biofuel crop for the production of ethanol [2]. It is a perennial grass, a member of the *Poaceae* family and *Andropogoneae* tribe, which includes maize and sorghum. Cultivated sugarcane is derived from inter-specific hybridisation between, in the main, two polyploid species *S. officinarum* L. and *S. spontaneum* L which have different basic chromosome numbers. The resultant cultivars are complex aneu-polyploids with chromosome numbers of 2n = 100-120. In the production of modern cultivars derived from the initial hybrid, the 2n transmission that occurs from *S. officinarum* in the primary cross has been used to advantage. Backcrossing to *S. officinarum* or other cultivars recovers the high sugar phenotype inherited from *S. officinarum* while retaining the disease resistance and good ratooning of the wild *S. spontaneum* parent. The preferential outbreeding, highly heterozygous and predominantly autopolyploid genetics of sugarcane are all factors that have hindered the development of a comprehensive genetic map.

Classic cytological studies using *in situ* hybridization have determined that *S. officinarum* is an octaploid (2n = 8x = 80) with a basic chromosome number of 10, the same as its closest known diploid relative sorghum [3]. *S. spontaneum* has varying ploidy levels (2n = 40-180) but has a basic chromosome number of x = 8 [3]. Genome *in situ* hybridisation has determined that commercial cultivars contain approximately 80% *S. officinarum* chromosomes, 10-15% *S. spontaneum* chromosomes and approximately 10% recombinant chromosomes [4, 5]. Q165 is an Australian cultivar no longer grown commercially. It has 110 chromosomes of which 82-83 (75%) are inherited from *S. officinarum,* 15-17 (15%) are inherited from *S. spontaneum* and 11-12 (11%) are recombinant chromosomes [3]. These are similar in the proportions found in most varieties studied to date [5].

Sugarcane has a large genome (10 Gb) [6] and in the absence of a genome sequence, a high-density genetic map is a valuable tool to understand the genetic and genomic organisation of this complex polyploid crop. Its autopolyploid nature with mostly random pairing plus high inbreeding depression has limited the production of more common experimental mapping populations such as double haploids or recombinant inbred lines. The complication of the coexistence of single dose (SD) and multi dose alleles and irregular chromosome numbers in the various homo(eo)logy classes due to aneuploidy has restricted genetic mapping. With the realisation that SD markers can be used to generate genetic maps in polyploids [7] genetic maps were developed in sugarcane using a population of full-sib (F_1_) individuals (pseudo-test cross strategy) [8] with SD markers segregating 1:1 or by using a population created by selfing an individual and mapping SD markers segregating 3:1. Initially mapping was carried out in the ancestral species *S. spontaneum*[9–11] and *S. officinarum*[12, 13]. More recently cultivars of hybrid origin have been mapped [14–20]. All of the genetic maps that have been generated for sugarcane to date have had low genome coverage and limited information on genome organisation. One of the main reasons for this has been the limited number of markers mapped. In most cases fewer than 1000 have been mapped due to the complexity of marker generation in such a complex polyploidy and the high cost of generation of large numbers of mappable markers. Many markers of different dosage are generated in sugarcane but segregation of markers of greater than double dose in the 200-300 size populations means that accuracy of marker placement is low [7].

The development of Diversity Array Technology (DArT) [21] for sugarcane has combined the low-cost high-throughput properties of the DNA microarray platform with the ability to identify various types of DNA polymorphisms [22].

This paper reports the generation of a comprehensive sugarcane genetic map of Q165, an Australian sugarcane incorporating 2267 markers generated from DArT, amplified fragment length polymorphism (AFLP), simple sequence repeats (SSR), single nucleotide polymorphism (SNP), restriction fragment length polymorphism (RFLP) and random amplified polymorphism (RAPD) markers. This large number of markers allowed the majority of the LGs to be placed into the 8 homology groups which is consistent with the basic chromosome number of the ancestral species *S. spontaneum* and the lowest basic chromosome number identified in the *Saccharum* genus.

## Results

### Generation of mapping data

To generate the maximum number of polymorphic markers using the sugarcane DArT array, the DNA from each of the F_1_ progeny was digested with several different restriction enzyme combinations. DNA from each combination was then screened separately across the sugarcane DArT array [22]. Using these separately screened enzyme combinations, a higher number of polymorphic markers was generated. In total 1555 markers were scored as present/absent of which 726 were generated from a *Pst*I/*BstN*I digest, 640 from a *Pst*I/*Hpa*II digest and 189 from a *Pst*I/*Taq*I digest. Of the 1555 DArT markers generated 263 were identified by more than one enzyme digest and had the same segregation pattern. The remaining 1246 unique markers included 1018 (82%) which were SD and used to generate the linkage map. *Pst*I/*BstN*I generated 580 (80%) SD markers, *Pst*I/*Hpa*II generated 528 (83%) and *Pst*I/*Taq*I generated 163 (87%).

In total 3475 markers were scored. Of these, 598 were SSR markers of which 473 were SD, 1062 AFLP markers of which 770 were SD, 184 SNP markers of which 153 were SD, 43 RAPD markers of which 26 were SD; and 33 were RFLP markers of which 27 were SD (Table 1; Additional file 1). The majority of the segregating markers inherited from Q165 were SD, and segregating 1:1 (present:absent) in the population (Tables 1 and 2).

**Table 1:** **Total number of single dose (SD), double dose (DD), triple dose (TD) and bi-parental SD markers (3:1 markers) scored in the parents of the mapping population**

	sugarcane cultivar (hybrid)	***S. officinarum***
	Q165	IJ76-514
**Chromosome no.**	110	80
**Number of markers**	3182	1168
**Number of SD markers**	2467	529
**Number of DD markers**	486	444
**Number of TD markers**	17	1
**Number of 3:1 markers**	256	
**Total number of LG**	160	66^1^
**No. of SD markers in map**	2267	-

^1^The addition of DArT markers did not provide the same magnitude of improvement to the IJ76-514 linkage map and the data is not shown

**Table 2:** **Number of markers generated with the different marker systems and number of single dose (SD) markers (as a percentage of the total number)**

Markers	Q165	SD (%)
**DArT**	1246	1018 (82%)
**AFLP**	1062	770 (73%)
**SSR**	598	473 (79%)
**SNP**	184	153 (83%)
**RAPD**	43	26 (60%)
**RFLP**	33	27 (82%)
**Total**	3182	2467

### Genetic map construction

**Figure 1:**
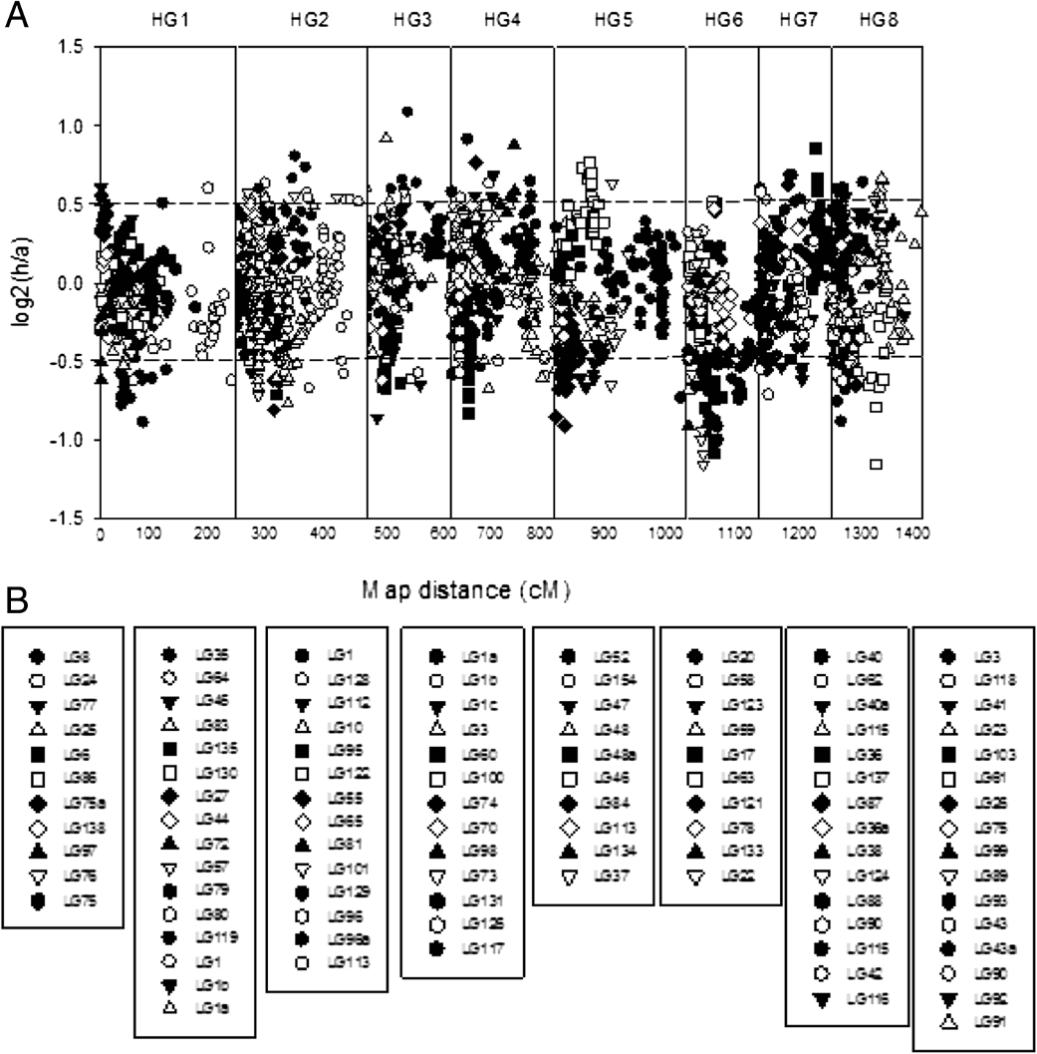
**Scatter plot representing the distribution of marker segregation distortion within each LG within the HGs with each dot representing one molecular marker.** The vertical solid lines distinguish the 8 HGs which represent the 8 basic chromosomes, along the total map distance (x axis). The y axis is the log2 value of the ratio of the number of individuals carrying the marker compared to the number of individuals not carrying the marker. Markers outside the two horizontal dotted lines are significantly skewed as calculated by the Chi-square test at p = 0.05.

**Figure 2:**
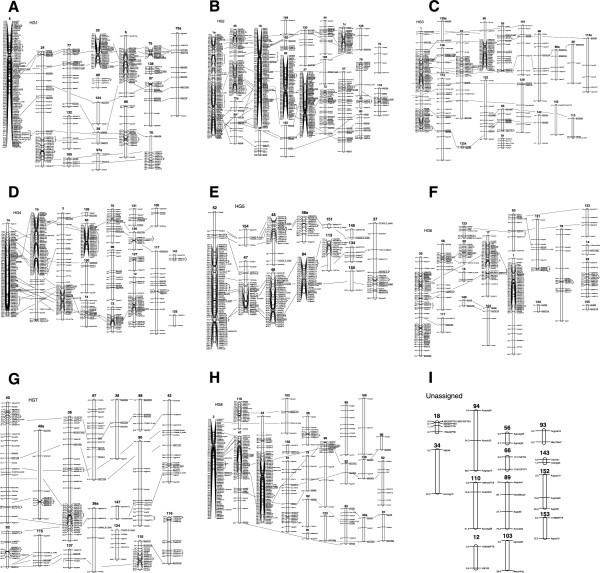
**A genetic linkage map of Q165 based on single dose markers. (A)** The LGs are formed into the 8 HGs using the alleles identified from SNP, SSR and RFLP markers, HG1, **(B)** HG2, **(C)** HG3, **(D)** HG4, **(E)** HG5, **(F)** HG6, **(G)** HG7, **(H)** HG8 and **(I)** unassigned LGs. A sugarcane genetic linkage map for the cv. Q165 generated from 227 individuals from a cross between Q165 and IJ76-514. The numbers on the left of the LG are the cumulative genetic distances in Haldane CentiMorgans. Marker names are shown on the right. The LGs are grouped into HG1 to HG8 and unassigned LGs using the alleles identified from SNP, SSR and RFLP markers. Allelic markers are in square boxes and the dotted lines represent the alignment between the LGs. The brackets are around DArT markers that are only different due to missing data.

The genetic map was constructed using 227 progeny generated from a cross between Q165 an old Australian cultivar and IJ76-514 a *S. officinarum* accession using the pseudo testcross approach. Of the 3475 markers scored that were present in Q165 and absent in IJ76-514, 2467 (78%) were SD in Q165. The majority of SD markers were DArT, AFLP and SSR markers although 184 EST SNP markers were also scored in this population (Table 2). Of the 2467 SD markers used for mapping, 967 were present in and used to generate the map reported in [15]. Although the markers were selected as SD markers segregating 1:1 in the progeny a chi square value was used that allowed inclusion of SD markers with moderate segregation distortion (skewed SD markers) but excluded likely double dose markers as in [23], as it is well known that segregation distortion is common in plants. The percentage of skewed markers varied from homology group (HG) to HG with 18% of markers skewed in HG7 and 37.5% of markers skewed in HG5 (Figure 1). Initially for map construction, only the markers that were present in greater than 188 progeny were used to generate a framework map. A number of the DArT markers (335 of the 1018 SD markers) were only scored in 94 individuals, and these markers were added to the map after initial map construction using markers scored across the whole population. The 2467 SD markers formed 160 linkage groups containing 2267 markers, leaving 200 (8%) unlinked markers (Table 3, Figure 2).

**Table 3:** **The number of each type of marker mapped within each homology group (HG) of Q165 and the number of linkage groups (LGs) formed within each (HG)**

HG	No. LG	No. AFLP	No. DART	No. SSR	No. SNP	No. RFLP	No. RAPD	Total number of markers mapped	Length of HG in cM	Marker density (cM)
**1**	17	82	125	60	13	1	1	282	1253.4	4.4
**2**	23	139	198	105	25	0	8	475	1818.0	3.8
**3**	20	77	75	52	11	2	1	218	1243.6	5.7
**4**	18	108	125	50	13	5	4	305	1210.7	3.9
**5**	13	71	94	32	24	1	0	222	846.6	3.8
**6**	18	69	111	34	13	2	3	232	678.3	2.9
**7**	16	62	90	27	12	8	5	204	1020.5	5.0
**8**	22	98	132	49	15	0	2	295	1319.2	4.4
**Unassigned**	13	19	10	3	0	0	2	34	384.1	10.9
**Total**	160	721	976	418	124	19	25	2267	9774.4	4.3

### Assembly of Saccharum homo(eo)logous groups (HGs)

As sugarcane is an autopolyploid it contains many copies of each chromosome. In order to determine the genome structure of Q165 the linkage groups (LGs) were placed into HGs using a number of different strategies. Firstly using the multi-allelic loci detected by single SSR, RFLP or SNP markers, secondly using sequence information from these markers and thirdly using repulsion phase analysis. The majority of the multi-allelic markers were from SSRs but a major problem encountered with the SSR markers was that only 47% of the 116 SSRs derived from sugarcane that were screened across the map, were specific to one homology group. Thirty-five of the SSRs had only one SD segregating marker and 26 (22%) mapped to more than one homology group. For the 43 SSRs derived from sorghum that were mapped, only 8 (19%) were specific to one homology group and the majority (27 markers, 63%) only generated one SD marker. Nevertheless, of the 160 linkage groups formed, 147 (92%) could be placed into HGs. These HGs contained from 13 to 23 LGs, with the LGs varying in length from 1.3 cM (consisting of two markers), to 232.7 cM (consisting of 81 markers) (Table 3, Figure 2). Coverage within the homology groups varied from HG2 with a total map length of 1818 cM to HG6 with a total map length of 678.3 cM (Table 3). HG 2 also had the most markers mapped with 475 markers compared to HG7 which contained the least number of markers at 204 (Table 3).

### Chromosome pairing within HGs

To determine whether there is preferential pairing behaviour within Q165 we examined linkage in repulsion of each marker with all other markers in the map. A total of 30 LGs were linked in repulsion with other LGs. The amount of repulsion detected varied between HGs, with HG6 having 7 LGs (33%) linked in repulsion compared to HG3 where no repulsion was detected (Table 4). In the HGs where repulsion was detected, the number of LGs involved varied from 2 to 7. Out of the 30 LGs linked in repulsion, 6 pairs of LGs were only found in repulsion to each other. The other 18 LGs were in repulsion with more than one other LG. This indicated that in most cases there was preference in pairing between a small number of LGs. A difference ratio was calculated to compare how the genetic distances of the LGs within a HG varied between HGs [24]. One high coverage LG was selected per HG to compare to all other LGs within the same HG (Table 5). The average difference ratio varied from 0.13 for HG3 to 0.86 for HG6.

**Table 4:** **Repulsion analysis between LGs within HGs**

HG	Number of LGs in repulsion	Number of LGs with no repulsion detected	Percentage of LGs with repulsion	LGs in repulsion	LOD score of linkage
1	3	14	18%	HG1LG8-HG1LG25	7
				HG1LG8-HG1LG6	7
2	5	18	22%	HG2LG1a-HG2LG27	7
				HG2LG1b-HG2LG1c	8
				HG2LG1a-HG2LG35	7
				HG2LG1c-HG2LG35	8
				HG2LG72a-HG2LG72	5
3	0	20	0		
4	2	16	11%	HG4LG1c-HG4LG1a	6
5	5	8	38%	HG5LG47-HG5LG46	12
				HG5LG52-HG5LG46	7
				HG5LG48-HG5LG48a	10
				HG5LG52-HG5LG47	7
6	7	11	33%	HG6LG20-HG6LG58	5
				HG6LG20-HG6LG1	5
				HG6LG20-HG6LG17	5
				HG6LG20-HG6LG59	5
				HG6LG58-HG6LG59	7
				HG6LG17-HG6LG59	7
				HG6LG16-HG6LG1b	6
7	4	12	25%	HG7LG42-HG7LG36	6
				HG7LG36-HG7LG36a	8
				HG7LG36-HG7LG38	8
8	4	18	18%	HG8LG41-HG8LG23	5
				HG8LG92-HG8LG93	5

**Table 5:** **The difference ratio of genetic distance in the common marker intervals within the sugarcane HGs between the selected LG A and the other LGs within the same HG B**

HG	LG	No. of shared marker intervals	LG _***max***_ (cM)	LG _***i***_ (cM)	∑ | ***LG***_***max-***_ LG _***ik***_ | (cM)	(LG_***max***_ + LG_***i***_) (cM)	Difference ratio
**HG1**							
LG8	LG24	10	747.7	310.6	437.1	1058.4	0.4
LG8	LG77	10	463.7	233.6	230.1	697.3	0.3
LG8	LG25	6	38.3	63.5	25.2	101.9	0.3
LG8	LG6	10	262.3	248.6	13.7	510.9	0.0
LG8	LG75	3	173.3	96.3	76.9	269.6	0.3
LG8	LG97	6	81.7	60.2	21.5	141.9	0.2
**HG2**							
LG1a	LG1b	15	1776.3	360.0	1416.3	2136.3	0.7
LG1a	LG1c	10	96.4	25.2	71.2	121.6	0.6
LG1a	LG27	6	58.4	83.2	24.8	141.6	0.2
LG1a	LG35	6	268.0	136.7	131.3	404.7	0.3
**HG3**							
LG1	LG55	1	37.1	29.5	7.6	66.6	0.1
LG1	LG10	3	170.4	84.2	86.2	254.6	0.3
LG1	LG128	1	1.0	1.1	0.1	2.1	0.1
LG1	LG112	1	21.3	24.1	2.8	45.4	0.1
**HG4**							
LG1a	LG3	6	434.7	278.7	155.9	713.4	0.2
LG1a	LG73	6	6.3	35.7	29.3	42.0	0.7
LG1a	LG131	1	7.6	4.7	2.8	12.3	0.2
LG1a	LG74	3	7.2	3.5	3.7	10.7	0.3
LG1a	LG1c	1	0.9	1.8	0.8	2.7	0.3
**HG5**							
LG46	LG47	3	11.3	2.4	8.9	13.7	0.6
LG46	LG84	1	8.9	29.4	20.5	38.2	0.5
LG46	LG52	1	12.7	14.1	1.5	26.8	0.1
**HG6**							
LG20	LG140	1	6.3	0.5	5.9	6.8	0.9
LG20	LG104	1	5.3	0.5	4.9	5.8	0.8
LG20	LG144	1	7.7	0.5	7.3	8.2	0.9
**HG7**							
LG40	LG87	1	12.1	4.8	7.3	16.9	0.4
**HG8**							
LG41	LG61	1	27.7	36.4	8.7	64.1	0.1
LG41	LG93	1	17.1	24.6	7.5	41.7	0.2

### Intrachromosomal rearrangements within the homology groups

#### HG1

HG1 contained 17 LGs which were grouped using 14 multi-allelic SSRs and 4 multi- allelic SNP markers. All of these multi-allelic markers were only mapped in HG1; there was only one allele from an SSR that mapped to another HG (Figure 2A). In general the LGs within the HG were syntenic to each other with the order of the markers highly conserved. Only HG1LG97 appeared to have a change in marker order at the end of the LG compared to other LGs within the HG with markers between M77i and M8a inverted.

#### HG2

This was one of the largest HGs with a total of 475 markers and the longest total length of 1818 cM (Table 3 Figure 2B). There were 30 multi-allelic markers with a range of alleles from 2 to 9 in this HG. Combined with the sequence information from markers with known sequence this produced a high coverage HG. The order of markers for the lower coverage linkage groups was fairly consistent with minor order changes probably as a result of missing data. There were five LGs (HG2LG1a, HG2LG35, HG2LG1b, HG2LG83, HG2LG27) with extensive rearrangements which appear to be recombinant LGs. These chromosomes are on average 52% longer than the rest of the LGs within this HG. This HG group contains the largest number of LGs and from the SSR markers there are two distinct sets of smaller LGs that align to these larger LGs. One set of LGs containing SSRs Msscir 17, Msmc1604, Msscir 44, Msmc 1825 and Msscir 46 and the other set of LGs containing Msscir28, Msmc336, Msscir 12, Msc851. This indicates that this HG is composed of *S. spontaneum* chromosomes aligned to two sets of *S. officinarum* chromosomes in agreement with the basic chromosome number of these progenitor species.

#### HG3

HG3 contained 218 markers with a total length of 1243.6 cM and an average distance of 5.7 cM between markers. This HG contained 20 LGs which were aligned with 17 multi-allelic SSRs and SNP markers (Figure 2C, Table 3). The overall order of markers was largely consistent between LGs.

#### HG4

HG4 contained a total of 308 markers covering 1217.2 cM which gave an average distance of 3.9 cM between markers (Figure 2D, Table 3). This HG contained 18 LGs assigned to the HG group using 19 multi-allelic markers with three densely mapped LGs and a number of smaller LGs. Marker order was maintained across the majority of linkage groups although some changes were seen. For example msc274 on HG4LG73 was in a different position relative to HG4LG1.

#### HG5

HG5 contained 222 markers that covered a distance of 846.6 cM with an average distance of 3.8 cM between markers (Figure 2E, Table 3). This group contained 13 LGs assigned to the HG with 8 multi-allelic markers. The LGs ranged from 225.8 cM with 66 markers to 5.1 cM with 2 markers.

#### HG6

HG6 contained 232 markers on 18 LGs which covered 678.3 cM with an average distance between markers of 2.9 cM (Table 3). This HG contained 7 multi-allelic markers and in general the marker order was consistent between LGs. HG6LG20 contained 6 markers at the end of the linkage group that also aligned to HG3. This appears to be a translocation between the two HGs. There is a 10 cM gap between the groups of markers but the groups were formed at a LOD of 20 giving strong evidence that the linkage is real (Figure 2F).

#### HG7

HG7 contained 204 markers which covered 1020.5 cM with an average distance between markers of 5 cM. This HG consisted of 16 LGs. Again there were fewer multi-allelic markers but from the 12 that were present in the group the marker order was consistent between LGs (Figure 2G).

#### HG8

HG8 contained 295 loci which covered 1319.2 cM giving an average of 4.4 cM between markers. This homology group contained 23 LGs formed using 9 multi-allelic markers. This HG contained a larger number of low coverage LGs but in general marker order was consistent between LGs (Figure 2H, Table 3).

### Comparison to the R570 genetic linkage map

Using common SSR markers, this current Q165 map can be partially aligned to the R570 genetic map [16]. For most of the HGs in the Q165 map each HG aligns to a single HG in the R570 map [16, 17] (Table 6). The major difference was that HG5 in the Q165 genetic map was missing in the R570 genetic map. There were only 3 SSRs in common between the Q165 HG5 and the R570 map and in all 3 cases, alleles from these SSRs mapped to two HGs. The SSRs markers Msscir47 and Msscir54 mapped to both HG5 and 8 in the Q165 map and to HG VI in R570; the latter aligns to HG8 in the Q165 map. The third marker (Msscir34) mapped to HG5 and 3 in the Q165 map and mapped to HGI in the R570 map; HG1 aligns to HG4 in the Q165 map. These incongruous markers could be explained by the mapping of duplicate loci in the two different cultivars which are polymorphic at different loci. Multi allelic markers that map to more than one locus can create problems for the assignment of LGs to HGs in sugarcane, but it is also possible that in a complex polyploid like sugarcane there are differences in chromosome composition between cultivars. Multi-locus markers are common in sugarcane; in the present study 22% of the SSRs mapped in Q165 mapped to two different loci. This phenomenon has also been observed in diploids [25].

**Table 6:** **Alignment of Q165 HGs with R570 HGs** [14, 16] **using the SSR markers in common**

HG Q165	HG R570 [13, 14]	Number of SSR markers in common	Number of SSR markers used to align the genetic maps	Number of incongruous markers
HG1	VII	5	5 (20)^1^	0^2^
HG2	VIII	7	7 (27)	0
HG3	II	8	7 (14)	3 (6)
HG4	I	5	4 (7)	1 (4)
HG5	-	-	-	-
HG6	IV	1	1 (1)	0
HG7	III	4	3 (8)	1 (3)
HG8	VI	7	6 (16)	3 (5)

^1^Total number of alleles in brackets which were mapped from the SSRs in common

^2^The number of SSRs and alleles in square brackets that map to different HGs.

## Discussion

### Sugarcane DArT markers

This is the first sugarcane genetic map published which contains DArT markers and our results demonstrate that sugarcane DArT markers are of high quality and can be used to substantially enhance an existing genetic linkage map. The DArT markers generated here integrated into the existing linkage map, demonstrating that they behave in a Mendelian manner. There was a higher level of redundancy observed for the DArT markers than for any of the other marker types but the DArT array used in this study was not filtered for redundancy. In total, 50% of the DArT markers were clustered within 10 cM regions across the genetic map but the location of these clusters corresponded with the location of clusters of other types of markers. This has been seen in previous DArT genetic maps for sorghum and oat [26, 27]. These high density clusters could correspond to the centromeric regions of chromosomes where large physical distances correspond to small genetic distances, a feature that has been seen before in sorghum [28]. In sorghum, the closest diploid relative of sugarcane, the pericentromeric heterochromatic regions of the chromosomes have been shown to have much lower rates of recombination (~8.7 Mbp/cM) compared to euchromatic regions (~0.25 Mbp/cM) [29]. It is also possible that some clusters represent additional regions where chromosomal rearrangements have occurred or where there is introgression from *S. spontaneum* causing a higher frequency of polymorphism and hence mapped markers.

### Q165 genetic map

This genetic map is a substantial improvement on previously published maps with more than twice as many markers compared to any other sugarcane genetic map. The high number of markers of known sequence contributed by the DArT, EST-SSR, and SNPs, allowed the majority of the LGs (92%) to be condensed into eight HGs that align to the basic chromosome number of *S. spontaneum*[3, 4]. The 160 LGs had a cumulative map length of 9792.7 cM and an average marker density of 4.3 cM. This current linkage map increased the total map length by 734.4 cM and decreased the average marker density from 8.43 cM in the previous Q165 map [13] to 4.3 cM. It is known from cytogenetic analysis that Q165 has 110 chromosomes [5]. In this study, the number of LGs greatly exceeds this number of chromosomes although 57 of the LGs are small with 3 or fewer markers (34%). This result gives an indication of the uneven coverage of markers across the genetic map which has been seen in all of the current published maps, whether generated from selfed populations [16, 19] or bi-parental crosses [17, 18, 20]. Sugarcane cultivars have over 100 chromosomes and are all derived from an initial hybrid between the two different species, *S. spontaneum* and *S. officinarum* followed by backcrossing to the high sugar *S. officinarum* or other cultivars. As a result, the background *S. officinarum* part of the genome has lower levels of polymorphism than the *S. spontaneum* part of the genome. In addition, it is well documented that 2n transmission of chromosomes occurs in interspecific crosses of sugarcane [30]. The introgressed *S. spontaneum* parts of the genome are the polymorphic regions and in part are responsible for the high density marker areas of the map. The high polyploidy of sugarcane means only a proportion of the markers that are polymorphic can be mapped. Any regions or marker alleles present at greater than two doses can not be accurately mapped with the present population size. The resulting Q165 map has the largest number of markers of any sugarcane linkage map published to date, with 2267 markers mapped across the genome, more than doubling the previous number of markers. However, this still only generates an average of 21 markers per chromosome.

### Marker order

Marker order was in good agreement between LGs within HG1, 4, 6 and 7 (Figure 2). Inconsistencies were observed in groups of markers that were closely linked. Inversion is a common feature of closely spaced markers [25] and could be real, or due to error, or could be explained by the statistical uncertainty of orders at the cM-scale that is inherent in large data sets. In contrast, marker order in HG2 and 8 was less consistent and could be explained by the fact these two groups contain translocated chromosomes. HG3 and 5 also appear to have simple translocations with marker order mostly maintained between LGs within a HG. Translocations have been identified in other polyploids; including wheat, an allopolyploid which has the well documented translocations between 4A, 5A and 7B [31, 32].

### Chromosome pairing

In total, repulsion was detected between 30 (19%) linkage groups, similar to the level detected in R570 where 18 LGs (21%) were involved in preferential pairing [17]. Analysis of all pairing frequencies determined that complete disomic behaviour was not present, rather the LGs preferentially paired and often within groups of 3 or 4 LGs. A cytogenetic study of Q165 demonstrated that its chromosomal make-up is 15-17 (15%) *S. spontaneum* chromosomes, 11-12 (11%) recombinant chromosomes [5] and 82-83 (75%) S*. officinarum* chromosomes. In total, Q165 contains 26-29 chromosomes with complete or partial *S. spontaneum* origin which is a similar number to the 31 LG identified with preferential pairing in this study. It is possible that the LGs displaying preferential pairing are the ones that are inherited from *S. spontaneum*. To determine how similar LGs were within a homology group, a difference ratio in genetic distance was calculated between LGs within a HG where marker intervals were shared [24, 25, 33]. The number of shared intervals varied between HGs and the accuracy of the difference ratio is probably higher in HG1-4 due to the larger numbers of shared intervals (Table 5). Combining this information with the preferential pairing results (number of LGs in repulsion) showed that HG3 had an average difference ratio of 0.13 and no preferential pairing, whilst HG6 had a ratio of 0.86 with 7 LGs involved in pairing. There was a positive correlation between the difference ratio and the percent of LGs detected in repulsion within a HG (Additional file 2) with an R^2^ = 0.44. The results indicate that within a HG group, as expected, there are two sets of LGs, each likely to be inherited from a different species. Furthermore, the HGs where these two sets of chromosomes are more distinct, for example HG6, showed more preferential pairing within and not between the two sets of LGs. In contrast, for HG3 where the LGs are more similar to each other, there is no preferential pairing.

### Comparison to R570

The majority of the Q165 HGs could be aligned with the R570 HGs using a total of 111 allelic makers from 37 SSRs (Table 6). The Q165 map forms 8 HGs aligning to the basic chromosome number of *S. spontaneum*. In the case of HGs 2 and 8 for Q165 and VIII and VI in R570 there appear to be more LGs than in the other HGs [17] Table 6. This could be because two sets of *S. officinarum* chromosomes are aligned with one set of *S. spontaneum* chromosomes which are the result of simple fusion [17]. This was first indicated in the R570 RFLP map [34] and verified in the more recent genetic map [17]. The R570 map is formed into 7 HGs and when aligned to the Q165 map appears to be missing HG5. HG5 in the Q165 map has 8 multi-allelic SSR and SNP markers and an additional 7 single SNP markers. From the location of the SSR markers Smc1047 and SM1420, HG5 appears to contain a translocation from HG2. It is possible that the R570 map lacks this HG5 because these duplicated SSRs have lead to the mis-assembly of sets of homologous LGs into the same HG [17]. It is likely that there will be complex structural differences between the basic chromosome sets of *S. officinarum* and *S. spontaneum* which may have lead to misassembly of HGs in both the R570 and Q165 maps. In the Q165 map there appears to be at least 3 LGs (HG5LG47, HG5LG46, HG5LG84) with this same translocation providing strong evidence for its existence (Figure 2).

## Conclusions

We have demonstrated that sugarcane DArT markers provide high quality markers that can be used to construct SD genetic maps in polyploid sugarcane. The high numbers of DArT markers generated in a single assay which are distributed over the whole genome offers a real advantage for a range of molecular breeding and genomics applications. As sugarcane cultivars have approximately 100-120 chromosomes, large numbers of markers are needed to generate a useful genetic map. The highly parallel and automated platforms used in DArT generation mean that the cost per data point is very low. The 2267 markers in this genetic map allowed 92% of the LGs to be placed into 8 HGs conforming to the basic chromosome number of the ancestral species, *S. spontaneum*. The large number of SSR markers on this genetic map provides the opportunity for the first time to compare genetic maps derived from different sugarcane cultivars. The cross-compatible markers used to generate the map provide the basis for further studies of comparative structural genomics within cultivated sugarcane varieties and within the *Saccharum* genus. The use of DArT markers will allow the development of consensus genetic maps in sugarcane which would improve genome coverage and allow integration with other genomic resources. A high coverage genetic map is essential for the correct assembly of the sugarcane genome sequence which a number of research groups are generating.

## Methods

### Plant material

The mapping population consisted of 227 progeny derived from a cross between a *S. officinarum* clone IJ76-514 (2n = 80) as the female parent and Q165 (2n = 110), an old Australian cultivar and elite parent.

### Marker generation

The marker data consisted of the original data from [15] to which were added over twice as many new markers generated with AFLP, SSR, RFLP, SNP and DArT markers. Generation of further SSR, and *Pst* I/*Mse*I AFLP marker data were as reported in the previous Q165 genetic map [15]. All SSR markers are named using the same convention as in [15]. An additional 43 SSR markers were added which were generated from sorghum (with prefix Txp) [35, 36]. The DArT data was generated as previously described [22]. To increase the number of polymorphic markers, the population was screened twice more across the sugarcane DArT array after cutting the DNA with an extra enzyme, *BstN* I or *Hae* III. The DArT markers were designed with the clone number followed by PTB for the BstNI digestion, PTH for the HaeIII digestion and PTP for the original *Pst*I digestion. DArT markers with a quality score of Q > 80% and a call rate of at least 80% were selected for mapping. The SNP data was generated using a Sequenom assay developed as previously described in [37]. The SNP markers were named with their gene index name and an allele number. Gene Index names correspond to the *Saccharum officinarum* Gene Indices at the Computational Biology and Functional Genomics laboratory at the Dana-Farber Cancer Institute (http://compbio.dfci.harvard.edu/tgi/cgi-bin/tgi/gimain.pl?gudb%20=%20s_officinarum). The 27 RFLP markers were generated as previously described in [38], and they were named with a clone number from a sugarcane cDNA library and the enzyme used for digestion. An additional two markers were derived from maize genomic cDNA prefix UMC [39].

### Genetic mapping

All marker systems produced monomorphic and polymorphic fragments. Each segregating band was scored independently as a dominant marker (presence vs. absence). As sugarcane is highly polyploid only SD markers were used for map construction, that is, markers that were present in Q165 and absent in IJ76-514. These markers displayed segregation ratios that did not significantly differ from 1:1 [7] at P = 0.05 by the Χ^2^ test [13]. The newly generated SD markers were added to the 967 existing markers from [15]. The new map was constructed using the software Joinmap 4.0 [40]. Marker groups were formed by two-point analysis at a LOD score threshold of at least 10 and a recombination fraction of 0.35. The linkage groups (LG) were then ordered using standard methods in JoinMap. The maps were generated in two steps. Initially only markers that were run on at least 188 progeny were used to generate a framework map, and then additional DArT markers that were only scored on 94 progeny were added to the framework map. LGs were then assembled into homology groups (HGs) using a minimum of two common markers.

### Investigation of chromosome pairing

Chromosome pairing was investigated by analysing markers in repulsion. The segregation data matrix was inverted then combined with the original data. A two point analysis was then carried out to identify linkage between the original data and the reverse phase data to identify pairs of markers in repulsion at a LOD ≤ 5. The distribution of these markers was analysed, and repulsion only accepted where large parts of the LG were involved, as artefactual repulsion is more likely to involve single pairs of markers [7, 41]. To determine how variation between the LGs within a HG affects chromosome pairing, a distance measurement of intervals between two individuals [24] as modified by [33], was used to compare the genetic distances between LGs within a HG to the most densely mapped LG within the same HG. The modified distance measure:


Where LG_*max*_ is the length (cM) of the *k*th shared marker interval on the most densely mapped LG within a HG and Lg_ik_ is the length (cM) of the *k*th shared marker interval on the *j*th LG within the same HG. The ∑ | LG_max_-LG_jk_| is the absolute value of the length difference of each shared marker interval within a HG between LGs LG_max_ and LG_i_ and LG_*max*_ + Lg_*i*_ is an additive value of all shared intervals for the *i*th LG within HG which is used to normalise the difference value ∑ | LG_max_-LG_jk_| [33]. These values were then averaged within a HG to give an average difference ratio.

## Electronic supplementary material

Additional file 1: **Q165 genetic map.** Excel spreadsheet containing a list of all map loci, and map positions in each HG. (XLSX 70 KB)

Additional file 2: **A graph of the correlation between number of LGs within a HG in repulsion and the average distance ratio calculated from Table** 5 **.** (DOC 48 KB)
