# Supplementary material for: A comprehensive genetic map of sugarcane that provides enhanced map coverage and integrates high-throughput Diversity Array Technology (DArT) markers
Source: BMC Genomics. 2014 Feb 24;15(1):152. doi: 10.1186/1471-2164-15-152 (PMC4007999; doi:10.1186/1471-2164-15-152)
Supplement: Supplementary file 2 — Additional file 2: A graph of the correlation between number of LGs within a HG in repulsion and the average distance ratio calculated from Table 5 . (DOC 48 KB) [file 12864_2013_7014_MOESM2_ESM.doc]

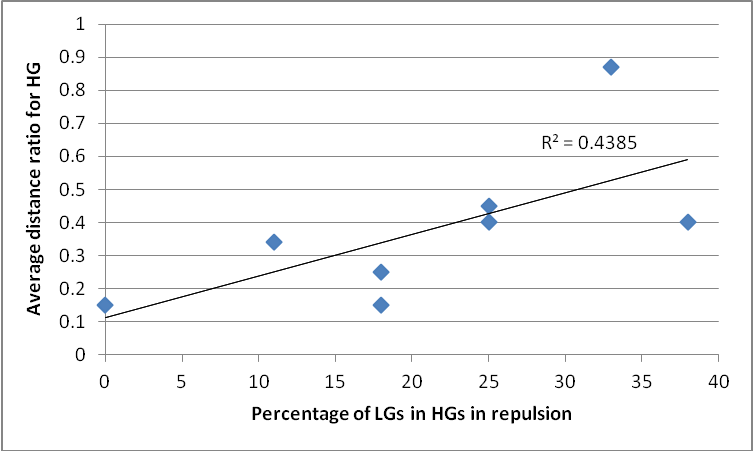


Correlation between percentage of LGs within a HG in repulsion and the average distance ratio calculated from Table 5.
